# Molecular and Immunological Characterization of Gluten Proteins Isolated from Oat Cultivars That Differ in Toxicity for Celiac Disease

**DOI:** 10.1371/journal.pone.0048365

**Published:** 2012-12-17

**Authors:** Ana Real, Isabel Comino, Laura de Lorenzo, Francisco Merchán, Javier Gil-Humanes, María J. Giménez, Miguel Ángel López-Casado, M Isabel Torres, Ángel Cebolla, Carolina Sousa, Francisco Barro, Fernando Pistón

**Affiliations:** 1 Departamento de Microbiología y Parasitología, Facultad de Farmacia, Universidad de Sevilla, Sevilla, Spain; 2 Instituto de Agricultura Sostenible (C.S.I.C.), Córdoba, Spain; 3 Hospital Virgen de las Nieves, Granada, Spain; 4 Departamento de Biología Experimental, Campus Universitario Las Lagunillas, Jaén, Spain; 5 Biomedal S.L., Sevilla, Spain; Ghent University, Belgium

## Abstract

A strict gluten-free diet (GFD) is the only currently available therapeutic treatment for patients with celiac disease (CD). Traditionally, treatment with a GFD has excluded wheat, barley and rye, while the presence of oats is a subject of debate. The most-recent research indicates that some cultivars of oats can be a safe part of a GFD. In order to elucidate the toxicity of the prolamins from oat varieties with low, medium, and high CD toxicity, the avenin genes of these varieties were cloned and sequenced, and their expression quantified throughout the grain development. At the protein level, we have accomplished an exhaustive characterization and quantification of avenins by RP-HPLC and an analysis of immunogenicity of peptides present in prolamins of different oat cultivars. Avenin sequences were classified into three different groups, which have homology with S-rich prolamins of Triticeae. Avenin proteins presented a lower proline content than that of wheat gliadin; this may contribute to the low toxicity shown by oat avenins. The expression of avenin genes throughout the development stages has shown a pattern similar to that of prolamins of wheat and barley. RP-HPLC chromatograms showed protein peaks in the alcohol-soluble and reduced-soluble fractions. Therefore, oat grains had both monomeric and polymeric avenins, termed in this paper gliadin- and glutenin-like avenins. We found a direct correlation between the immunogenicity of the different oat varieties and the presence of the specific peptides with a higher/lower potential immunotoxicity. The specific peptides from the oat variety with the highest toxicity have shown a higher potential immunotoxicity. These results suggest that there is wide range of variation of potential immunotoxicity of oat cultivars that could be due to differences in the degree of immunogenicity in their sequences.

## Introduction

Celiac disease (CD) is an autoimmune disorder with genetic, environmental, and immunological components as a consequence of sensitivity to gluten proteins present in cereals. The peptides produced in the digestive tract by the partial digestion of gluten from wheat, barley, rye and possibly oats, cause inflammation of the small intestine and villous atrophy. The prevalence of CD in Western countries is about one in 250 people, but estimates of up to 1% of the population have been made [1], [2]. The response is a consequence of deamidation of glutamine residues in peptides, resulting from activity of the tissue transglutaminase (tTG) in the gut mucosa. The modified peptides are able to bind to class II human histocompatibility leukocyte antigen (HLA) molecules DQ2 and DQ8, which stimulate T-cells, resulting in an inflammatory response in the small intestine that leads to flattening of the mucosa [3]. Currently, the only effective treatment for CD is to maintain a strict gluten-free diet throughout life. However, this is complicated as gluten is a ubiquitous additive in most sectors of the prepared-food industry.

Gluten is a complex mixture of polypeptides present in cereals such as wheat, barley, rye, and oats. In wheat, gluten consists of two fractions: an ethanol soluble one, termed gliadins, and the other insoluble termed glutenin [4], [5]. The homologous ethanol soluble fractions of barley, rye, and oats, are termed hordeins, secalins, and avenins respectively. The availability of full amino acid sequences has shown that this group of proteins has high content of proline and glutamine and, for that reason, has been termed prolamins [6].

The main immunogenic components of wheat gluten are the gliadins, a family of proteins characterized by their high content of proline and glutamine residues, 15% and 35% respectively [7]. Two monoclonal antibodies (moAbs), G12 and A1 [8], [9], were developed against 33-mer, a major immunotoxic peptide from α-2 gliadin [10]. These antibodies also recognize with high sensitivity other immunotoxic peptides from wheat, barley, and rye. Analysis of T-cell reactivity and detoxification proteins showed that the signal of these antibodies was correlated with the toxic potential of samples for celiac patients [8]. In those studies, the G12 antibody showed cross-reactivity that was used to detect avenin in oats, although with lower sensitivity than for the prolamins of wheat, barley, or rye. The monoclonal antibody G12 has three recognition epitopes along the sequence of the 33-mer. Thus, the lower sensitivity shown by the G12 antibody against prolamins from oats may be due to the lower affinity for the epitopes present in avenins.

Cultivated oats are hexaploid cereals belonging to the genus *Avena* L., which is found worldwide in almost all agricultural environments (reviewed by [11]). Recently, oats have been receiving increasing interest as human food, mainly because the cereal could be suitable for consumptions by celiac patients [12]. Oats have other nutritional attributes such as those derived from β-glucan content [13], or the protein amino acid composition [14].

The inclusion of oats in “gluten-free” foods is controversial, as previous studies have shown contradictory results on its toxicity. Some researchers claim that celiac patients can tolerate oats without signs of intestinal inflammation [12]. However, other studies confirm the toxicity of oats in certain types of celiac patients [15], [16]. More recently, the utility of the G12 antibody to identify potentially toxic oat varieties for celiac patients has been reported [17]. This finding allowed classification of oat varieties into three groups based in their degree of affinity for the G12 antibody: a highly recognized group, one of moderate recognition, and one with no reactivity [17]. The reactivity that T-cells isolated from celiac patients exhibited with three oat varieties (one from each of the classified groups) correlated directly with the moAb G12 reactivity. The diversity observed in the reactivity to the different oat cultivars suggests variations in the avenin composition, and therefore in the amount of immunotoxic epitopes similar to the 33-mer present in these varieties.

In comparison with wheat gliadins, the avenins have been little studied, and the number of full avenin genes present at the moment in the databases is limited and from few genotypes, so that the variability of avenin genes in oats is not well represented. With this background, the aim of the present work was to obtain further gene sequences from different toxic and non-toxic varieties of oats in order to provide more information on the structure of avenin genes and on the evolutionary relationships with the prolamins and glutenins of wheat and other cereals. It also would facilitate the identification of toxic epitopes described in other cereals that might be present in oats. Furthermore, these sequences could lead to the discovery of new undescribed toxic epitopes in cereals and explain why certain varieties of oats are toxic for celiac patients and others are not. In this work we demonstrate that oat grains have both monomeric and polymeric avenins, designated in this paper gliadin- and glutenin-like avenins. We found a direct correlation between the immunogenicity of the different varieties of oats and the presence of the specific peptides with a higher/lower potential immunotoxicity. Our results suggest that there is a wide range of variation in the potential immunotoxicity of oat cultivars that could be due to differences in the degree of immunogenicity in their sequences.

## Materials and Methods

### Plant material

Oats (*Avena sativa* L.) from cultivars designated OM719, OH727, OF720 and OP722 [17] and *Triticum aestivum* cv ‘Bobwhite 208’ (BW 208) were used in this work. Plants were grown in pot of 1 liter filled with a potting mixture of 1 part of Floragard Universal Potting Soil (FloraGard Product), 1 part of sand and 1 g/L of mixture of Osmocote Exact Standard (Scotts® Professional products) in a greenhouse with supplementary light to extend the photoperiod to a day/night regimen of 12/12. These cultivars were chosen based on their previously reported CD toxicity [17]. Cultivar OM719 was highly toxic, cultivar OH727 was moderately toxic and cultivars OF720 and OP722 were non-toxic [17].

In this work, the oat seeds were collected at 23 days post anthesis (DPA) for the amplification and cloning of gliadin-like avenin genes and at 0, 4, 8, 12, 20 and 28 DPA for the quantification of avenin genes expression.

### RP-HPLC

Oat samples and a wheat control were dehulled manually and ground in a Retsch MM200 (Retsch GmbH & Co., Haan, Germany) ball mill with stainless steel balls. Prolamins were extracted from oat and wheat flours using a modified classical Osborne procedure based on proteins solubility [5].

The gliadin-like avenin fraction from 100 mg of flour was extracted stepwise three times with 670 µL of 60% (v/v) ethanol, vortexing for 2 min at room temperature (RT), followed by incubation at RT for 10 min with shaking. Samples were centrifuged at 6,000× *g*. for 20 min, and the supernatants were collected and mixed together. The insoluble material from the previous step was used to obtain the glutenin-like fraction, which was extracted stepwise twice with 500 µL of 50% (v/v) 1-propanol, 2 M urea, 0.05 M Tris-HCl (pH 7.5) and 2% (w/v) DTT, vortexing for 2 min at RT and incubating for 15 min at 60°C with shaking. Samples were centrifuged at 6,000× *g*. for 20 min, and the supernatants were collected and mixed together. Finally, samples were filtered through a 0.45 µm nylon filter (Teknokroma, Barcelona, Spain).

Gliadin-like (100 µL) and glutenin-like (100 µL) extracts were applied to a 300SB-C8 reversed-phase analytical column (4.6×250 mm, 5 µm particle size, 300 Å pore size; Agilent Technologies) using a 1200 Series Quaternary LC System liquid chromatograph (Agilent Technologies) with a diode-array ultraviolet-visible detector (DAD UV-V), as described by [18]. The software, with some minor manual adjustment, handled the integration procedure automatically. Absolute amounts of gliadin and glutenin fractions were determined using bovine serum albumin (BSA; BSA ≥98%, fraction V. Sigma-Aldrich, St Louis,MO, cat. no. A3294) as protein standard. Three independent repetitions were carried out for each oat line. The ω-, α- and γ-gliadins for the ethanol-soluble fraction and the HMW and LMW subunits for the reduced-soluble fraction were identify in the RP-HPLC chromatograms by elution order and retention times according to [5].

**Figure 1:**
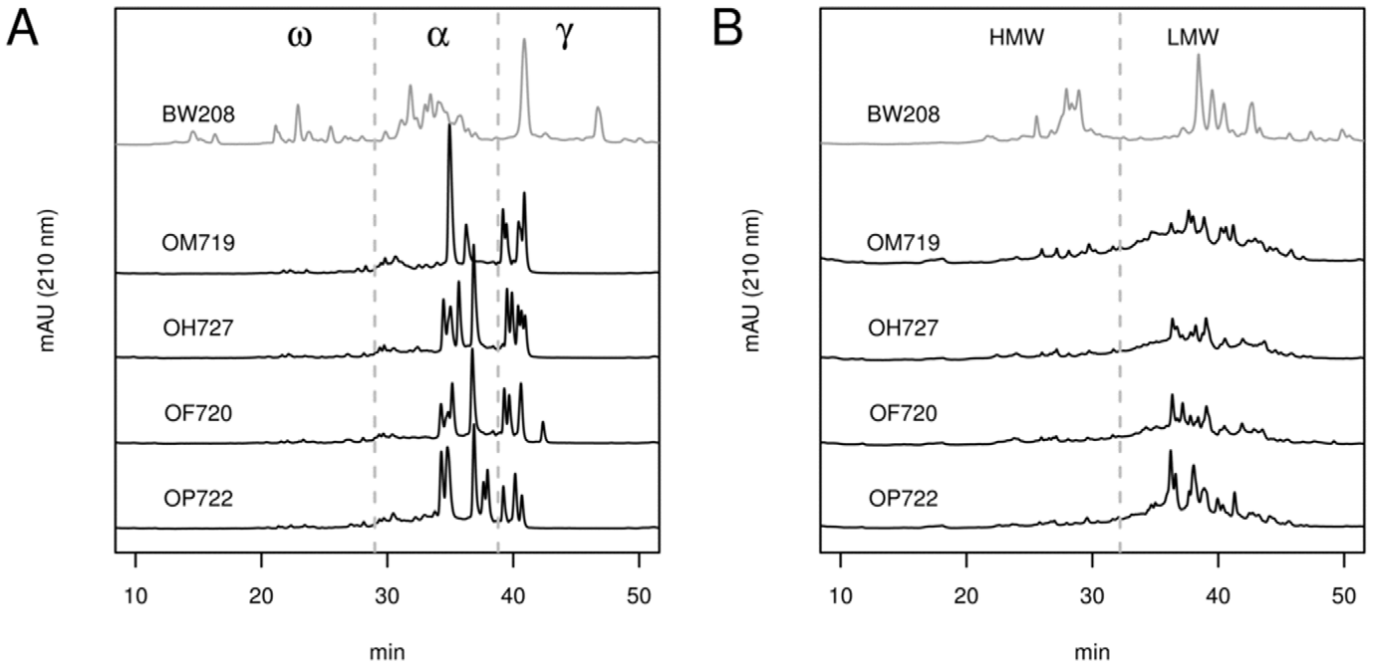
RP-HPLC chromatograms of prolamin extracts from different oat varieties and bread wheat (BW208). (A) Gliadin and gliadin-like extracts from four oat varieties and bread wheat used as reference. (B) Glutenin and glutenin-like extracts from oat varieties and bread wheat used as reference. ω, ω-gliadins and ω-gliadin-like fraction; α, α-gliadins and α-gliadin-like fraction; γ, γ-gliadins and γ-gliadin-like fraction; HMW, high-molecular-weight glutenin subunit (HMW-GS) and HMW-GS-like fraction; LMW, low-molecular-weight glutenin subunit (LMW-GS) and LMW-GS-like fraction. mAU (210 nm), milliunits of absorbance at 210 nm; min, retention time in minutes.

All analyses and plots were conducted with the statistical software R version 2.14.1 [19]. Data were tested for normal distribution using the Shapiro–Wilk test (function *shapiro.test*, package *stats*) [20], and for homogeneity of variances with the Levene's test (function *leveneTest*, package *car*) [20]. The differences between the oat lines were assessed using one way analysis of variance, followed by LSD (Least Significant Difference) multiple-comparison test (function LSD.test, package agricolae) [21]. Plots (Figure 1, Figure S1 and Figure S2) were generated with the function matplot (package graphics).

### RNA isolation and synthesis of cDNA

Oat seeds were collected in liquid nitrogen and stored at −80°C until use. Frozen seeds were pulverized under liquid nitrogen. Total RNA was isolated using the Trizol reagent (Invitrogen, Carlsbad, California, USA) with modifications of the company's instructions due the high polysaccharide content of the grains and treated with DNase I (RNase-free) (Ambion, Austin, Texas, USA) to eliminate any DNA contamination. For the gene expression analysis, RNA was purified using the RNeasy mini kit (Qiagen,Valencia, California, USA). First-strand cDNA was synthesized from 2 µg of total RNA, using oligo (dT) primer, random nanomers and M-MLV reverse transcriptase (Invitrogen, Carlsbad, California, USA) in 20 µL total volume according to the manufacturer's instructions. cDNA samples were diluted with an additional 80 µL of milliQ water.

### Amplification of gliadin-like avenin genes

Three primers, one forward and two reverse (AvenG-1F, AvenG-1R, AvenG-2R), were developed for the amplification of oat prolamin genes (Table 1). These primers were designed based on avenin-coding sequences/regions (GenBank accession no. DQ370180, M38722, M83381, J05486, M38721, CK780254, and CK780283). cDNA PCR amplifications with primer combinations AvenG-1F/AvenG-1R and AvenG-1F/AvenG-2R were conducted in 25 µL reaction volume consisting of 2 µL cDNA dilution, 0.2 mM dNTPs (Promega, Madison, Wisconsin, USA), 0.2 µM of each primer, 2 mM MgCl_2_, 1X PCR buffer and 2 units of *Taq* DNA polymerase (Bioline, Boston, Massachusetts, USA). The cycling parameters were 28 cycles of 94°C for 15 s, 68°C for 30 s (decreasing 0.5°C each cycle), and 72°C for 1 min, followed by 30 cycles of 94°C for 15 s, 56°C for 30 s, and 72°C for 1 min. The electrophoretic separation of amplified products was conducted on 1% agarose gel in 0.5X Tris-borate-EDTA (TBE) buffer.

**Table 1:** PCR primers used for cloning, characterization and quantification of avenin genes.

Primer	Description	Sequence (5′→3′)
AvenG-1F	Forward primer for avenin genes	ATGAAGAMCTTYCTCATCHTTGC
AvenG-1R	Reverse primer for avenin genes	TYAGAAGCCACYGAGMGGG
AvenG-2R	Reverse primer for avenin genes	TTAGCAACCACCAATGCC
AvenB-q1F	Forward for group A quantification	GGTCTCGTTCAGCCTCAGAC
AvenB-q1R	Reverse for group A quantification	GTCTGCATCGCTACCACCTT
AvenA2-q1F	Forward for group B quantification	TGAGCAACAACAGCCAATTC
AvenA2-q1R	Reverse for group B quantification	CTGTTGCACGAGGAACTGC
AvenA1-q1F	Forward for group C quantification	ATCCTGCGACAGGCCATCT
AvenA1-q1R	Reverse for group C quantification	GCTTGCACTACGCTGTGGAT

### Cloning and sequencing of PCR products and sequence analysis

The resulting products of PCR amplification were subjected to ligation into the pGEM-T Easy vector (Promega, Madison, Wisconsin, USA), and introduced into competent *Escherichia coli* (DH5α) cells by heat shock transformation. The positive colonies were amplified using M13 universal primers to identify the clones with an insert. The plasmid was isolated and purified using a QIAprep Spin Miniprep Kit from Qiagen (Valencia, California, USA) and used as a sequencing template. A minimum of three clones from each PCR product were sequenced. The sequence analysis was conducted using the MEGA4 and CLC Main Workbench 6.2 (CLC Bio website. Available:http://www.clcbio.com. Accessed 2012 October 16.) software, and the programs available in the NCBI and EBI network (NCBI and EBI websites. Available: http://www.ncbi.nlm.nih/gov/, http://www.ebi.ac.uk/. Accessed 2012 October 16).

### Amino acid composition

Protein sequences from *T. aestivum* of each prolamin group were searched in the NCBI protein database (NCBI protein database website. Available: http://www.ncbi.nlm.nih.gov/protein. Accessed 2012 January 21.) using the following keywords and filter: for γ-gliadin, (γ gliadin) AND “Triticum aestivum”[porgn:__txid4565]; for ω-gliadin, (ω gliadin) AND “Triticum aestivum”[porgn:__txid4565]; for α-gliadin, (α gliadin) AND “Triticum aestivum”[porgn:__txid4565]; for HMW-GS, (HMW) AND “Triticum aestivum”[porgn:__txid4565]; and for LMW-GS, (LMW) AND “Triticum aestivum”[porgn:__txid4565]. The percentage of each amino acid was calculated for wheat prolamin sequences and for the avenins sequences reported in this paper, using the bioperl script aacomp.PLS (Bioperl website. Available: http://www.bioperl.org/wiki/Bioperl_scripts. Accessed 2012 October 16.). The average percentage of amino acid composition over their respective sequences of each protein group was estimated.

All analyses and plots were conducted with the statistical software R version 2.14.1 [19]. The differences between amino acids percentages were assessed using one-way analysis of variance (function *anova*, package *stats*), and the multiple-comparisons were carried out with Tukey's HSD (Honestly Significant Difference) test to be more conservative in the conclusions and because the design was unbalanced (function *HSD.test*, package *agricolae*) [21]. Plots (Figure S3) were generated with the function *barplot2* (package *gplots*) [22]. We calculated the major amino acid in each group of prolamins. Amino acids considered major were those with a higher content and whose cumulative percentage was more than 50% of total amino acids of the protein sequences for each group.

### Quantitative real time PCR (qRT-PCR) of avenin genes

For the quantification of avenin gene expression we used the OM719, OH727, and OF720 varieties, one for each class of immunotoxicity as described previously by Comino et al., 2011. Three plants for each stage were analyzed using qRT-PCR. Developing grains were harvested at 0, 4, 8, 12, 20 and 28 DPA corresponding to growth stage numbers X6.1, Z7.02, Z7.08, Z7.1, Z7.5, and Z8.7 respectively, according to the Zadoks code [23]. Grains were harvested and bulked from the central part of heads from the primary tiller.

The gene-specific primers AvenB-q1F/AvenB-q1R, AvenA2-q1F/AvenA2-q1R, and AvenA1-q1F/AvenA1-q1R (Table 1) were used for the analysis of avenin gene expression. These primers were designed to amplify genes from each group of the neighbor-joining tree constructed using the full protein sequences. qRT-PCR was performed with a 7300 Real-Time PCR System (Applied Biosystems, Foster City, California, USA) using SYBR Green PCR Master Mix (Roche, Basel, Switzerland). The qRT-PCR reaction conditions for thermal cycling were following: 50°C for 2 min, 95°C for 10 min, followed by 40 cycles of 95°C for 15 s and 60°C for 1 min. Amplification specificity was verified with a heat dissociation protocol (melting curves in 60–95°C range) in the final step of PCR. All primer pairs showed a single peak on the melting curve, and a single band was visualized after separation by agarose gel electrophoresis.

### Peptic-trypsic-chymotrypsin digestion

The alcohol-soluble protein fraction was extracted from wheat and rice whole flour (positive and negative control, respectively) and subjected to peptic, trypsin and chymotrypsin sequential digestion according to [17].

### Synthesis of peptides

The peptides Q-14-5 (QQPFVQQQQQPFVQ), Q-14 (QQPFMQQQQPFMQP), Q-14-1 (QYQPYPEQQEPFVQ) and Q-14-3 (QQPFVQQQQPFVQQ), were supplied by Biomedal S.L. (Sevilla, Spain).

### Histological and serological analysis of subjects

Fourteen patients with biopsy-proven active CD were included in this study. The diagnosis of CD in the patients was determined by serological screening tests accompanied by biopsy of the small intestine according to the criteria of Marsh [24] and confirmation of a clinical response to gluten elimination from the diet. Subjects were prospectively screened for CD using anti-endomysial antibodies, tTG antibodies and CD-specific HLA (human leukocyte antigen) typing (Table 2). Venous blood was taken at the time of index biopsy. The study was approved by the ethics committee of the ‘Virgen de las Nieves’ Hospital, Granada (Spain), and informed consent was obtained.

**Table 2:** Clinical data of patients with celiac disease AATG, antitransglutaminase antibody, expressed as U/mL; AAEM, antiendomysial antibody; HLA, human leukocyte antigen; nd, no data.

Patient	Age (year)	Sex	Weight (Kg)	Height (Cm)	AATG (IgA)	AAEM	Atrophy grade (Marsh criteria)	HLA-DQB1
Celiac 1	4	Female	20	106	>200	+	IV	0201-0202
Celiac 2	4	Female	17.5	108	>200	+	III C	0201-0202
Celiac 3	1	Female	7.5	76	142	+	III B	nd
Celiac 4	3	Female	11	90	34	+	III A	0201-0603
Celiac 5	12	Male	49	151	>200	+	IV	0201-0503
Celiac 6	7	Male	23	123	>128	+	III A	0201-0301
Celiac 7	1	Male	10.5	82	>128	+	II	0201-0602
Celiac 8	5	Female	19	108	>128	+	III A	0201-0501
Celiac 9	10	Male	23.5	129	>200	+	IV	0201-0301
Celiac 10	2	Female	14	93	>128	+	III B	nd
Celiac 11	10	Female	24	132	>128	+	III B	0201-0202
Celiac 12	2	Female	13	92	16	+	III A	nd
Celiac 13	3	Male	13.5	91	91	+	III C	0201-0604
Celiac 14	5	Male	17.5	106	>200	+	III C	0201-0202

### Peripheral blood mononuclear cells (PBMCs) and cell cultures

PBMCs from patients with active CD who were on a gluten-containing diet were isolated from 6 mL of heparinized blood by Histopaque gradient centrifugation, and cultured at a density of 1×10^6^ cells/mL in RPMI-1640 culture medium. After 48 h, PBMCs were incubated with avenin, gliadin and rice prolamin peptides (50 µg/mL).

### Cell proliferation analysis

T-cell proliferation was determined after 48 h of incubation using the ELISA 5-bromo-2-deoxyuridine cell proliferation test (Millipore Chemicon, Temecula, California, USA). The stimulation index (SI) value was calculated by dividing the mean absorbance/10 at 450 nm after stimulation by the mean absorbance of T-cells exposed to the culture medium alone (negative control) and divided by 10. The proliferation of PBMCs exposed to the different peptides of oat prolamins was expressed as the mean fluorescence intensity 48 h after exposure.

### IFN-γ production

Supernatants from PBMC culture were collected after 48 h and stored at −80°C for IFN-γ determination using a commercial ELISA kit in accordance with the manufacturer's instructions (Thermo Scientific, Madrid, Spain). Standards were run on each plate. The sensitivity of the assay was <2 pg/mL.

### Statistical analysis of T-cell and IFN-γ assays

Each experiment was carried out in duplicate on separate days. Data are expressed as mean ± SD. All statistical analysis was performed with the STATGRAPHICS program. When the interaction was significant, the differences between groups were examined by one-factor analysis of variance (ANOVA). A Bonferroni-corrected Student t test was used to compare the individual means. A statistical probability of p<0.05 was considered significant.

## Results

### Gluten content and monomeric/polymeric protein distribution in oat kernels

In order to clearly describe the different oat protein fractions and avoid misunderstanding we will use the seed protein fractions of wheat as a reference. Hence, as the oat alcohol-soluble proteins have the same solubility features as the gliadins from wheat, throughout the article we will refer to these oat proteins as gliadin-like avenins. Similarly, the solubility of the oat reduced-soluble proteins is comparable to that of the glutenin proteins from wheat, therefore they will be designated as glutenin-like avenins.

Figure 1 shows chromatograms of alcohol-soluble (Figure 1A), and reduced-soluble fractions (Figure 1B). Plots show a representative chromatogram of each oat variety and of bread wheat used as a reference.

The gliadin components were distributed over nearly the whole elution range (Figure 1A) under conditions defined to optimize the resolution and reproducibility of gliadin separations. The different wheat gliadin types were eluted in the sequence ω-gliadins (retention time 10–29 min), α-gliadins (30–39 min) and γ-gliadins (40–50 min). In the case of oats, several minor components appeared in the hydrophilic range, although gliadin-like proteins comprised two major groups that were eluted close together in the hydrophobic range (34–43 min), at the same retention time as α- and γ-gliadins of wheat. The four oat varieties analyzed showed similar, but not identical, RP-HPLC patterns. Chromatograms showed between 7 and 11 major peaks in the varieties studied (Figure S1), with OH727 and OM719 having the highest and the lowest number of peaks, respectively.

An analogous analysis of the glutenin-like proteins of oats was carried out under the conditions optimized for wheat glutenin separation (Figure 1B). The glutenin proteins were distributed, as in the case of gliadins, over nearly the whole elution time. Wheat glutenins showed two main groups of peaks between the retention time 24–31 min and 35–50 min, corresponding to the high-molecular-weight glutenin subunit (HMW-GS) and low-molecular-weight glutenin subunit (LMW-GS), respectively [5], [25]. Major peaks were not detected in the range of HMW-GS for any of the oat varieties. On the other hand, all the oat varieties showed peaks in the elution range of the LMW-GS, although these peaks were less clearly defined than those for the gliadin-like fraction. Even so, the glutenin-like chromatogram profiles were different and easily distinguishable between oat varieties.

Because the glutenin-like fraction was extracted from the pellet resulting from a previous gliadin-like extraction, we compared the gliadin-like and glutenin-like chromatograms of each sample for the presence of common peaks (one representative example in Figure S2). Although some peaks overlapped in their retention times, most of the major peaks were unique in each of the fractions.

A quantitative analysis of the protein fractions was carried out by integrating the area of each individual peak present in the oat chromatogram region for α- and γ-gliadin-like proteins, and for the LMW-GS-like region of the glutenin-like fraction. Only regions with major peaks were considered. Data were transformed to micrograms of protein per milligram of flour, as shown in Table 3.

**Table 3:** Gliadin-like, glutenin-like, and total prolamin contents of oat varieties.

	Gliadin-like (µg/mg of flour)					
Line	α-	γ-	Total	LMW-GS-like (µg/mg of flour)	gli/glu	Prolamin (%)
OF750	7.51	3.54 **ab**	11.6	9.26	1.19	2.03
OH727	8.75	4.02 **a**	13.3	10.1	1.26	2.29
OM719	8.84	4.72 **a**	14.2	9.61	1.41	2.32
OP722	10.7	2.25 **b**	13.6	10.9	1.19	2.39

Gliadin-like and glutenin-like were determined by RP-HPLC. α, α-gliadin-like fraction protein content; γ, γ-gliadins-like fraction protein content; Total, total gliadin-like protein content; LMW, low-molecular-weight glutenin-subunit-like (LMW-GS-like) fraction protein content; gli/glu, ratio total gliadin-like content/total glutenin-like content. Values within the same column followed by the same letter are not significantly different at *P*<0.05 according to LSD test. Columns containing variables without letters mean that no significant differences were found between values.

The oat varieties did not show significant differences for the content of gluten protein fractions, except for the γ-gliadin-like fraction content. The multiple-comparisons analysis showed two groups for which the γ-gliadin-like content was statistically significant: in one group, line OP722 had the lowest amount of γ-gliadin-like proteins, and in the other, lines OM719 and OH727 showed a higher amount of γ-gliadin-like proteins. No differences in the total content of gliadin-like and glutenin-like proteins were found in the oat varieties.

### Cloning and structure analysis of gliadin-like avenins genes related to toxicity for celiac disease

Three primers, one forward and two reverses (AvenG-1F, AvenG-1R, AvenG-2R), were used to amplify the full-length ORF of avenin genes from four varieties of *A. sativa* with different levels of toxicity for CD patients. These primers were designed on the basis of the full avenin gene sequences available in the GenBank database (GenBank accession no. DQ370180, M38722, M83381, J05486, M38721, CK780254, and CK780283), and used to amplify the maximum amount of avenin sequences from the four oat varieties analyzed. Therefore, we inserted degenerate positions in the primers AvenG-1F and AvenG-1R to increase the range of avenin sequences amplified with these primers.

Sequences were amplified from cDNA (after reverse transcription from mRNA) using a DNA polymerase with proofreading capacity to minimize mistakes in the PCR during the incorporation of nucleotides. In total, 22 full-length sequences were obtained (Table 4): three from OF720, seven from OH727, eight from OM719, and four from OP722 (sequences are deposited in the GenBank database under accession numbers JX428830–JX428851). The ORF length of the sequences ranged from 546 bp to 846 bp, corresponding to 181 and 281 amino acids, respectively. The BLAST searches of all sequences using the *blastn* and *blastp* algorithms confirmed their identity with other avenins registered at the GenBank, and the homology with wheat gliadins.

**Table 4:** Avenin features.

			Number of amino acid residues				
Oat line	Sequence name	cDNA (bp)	Total	Nter	Rep	Cter	Cysteine
OF720	OF720-1A3	690	230	0	46	165	8
	OF720-AC2	603	201	4	46	132	9
	OF720-BC1	546	182	0	25	138	8
OH727	OH727-2A7	600	200	4	45	132	8
	OH727-2A8	672	224	0	73	132	8
	OH727-2A9	636	212	0	46	147	8
	OH727-2A10	846	282	4	73	186	8
	OH727-2A11	753	251	0	46	186	8
	OH727-AC1	603	201	4	46	132	8
	OH727-BC5	546	182	0	25	138	9
OM719	OM719-2A6	606	202	4	47	132	8
	OM719-AC2	639	213	0	47	147	8
	OM719-AC3	627	209	4	54	132	8
	OM719-BC4	570	190	0	32	139	9
	OM719-BC5	570	190	0	32	139	9
	OM719-BC6	570	190	0	32	139	9
	OM719-BC7	567	189	0	32	138	9
	OM719-BC8	546	182	0	25	138	9
OP722	OP722-3A1	645	215	4	46	146	8
	OP722-3A11	630	210	0	45	146	8
	OP722-AC2	630	210	0	44	146	8
	OP722-AC4	672	224	4	46	155	9

cDNA (bp), length in bases pair of cDNA; Nter, Amino-terminal conserved region; Rep, repetitive region; Cter, Carboxy-terminal conserved region.

**Figure 2:**
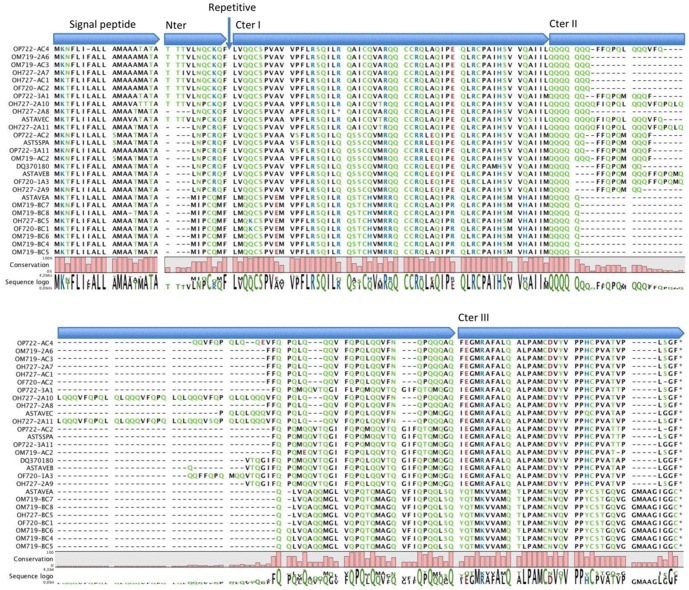
Alignment of avenins polypeptide sequences deposited at the GenBank and sequenced in this paper. Clustal-W algorithm (Thompson et al. 1994) was used to align the derived aminoacid sequences of avenins. The vertical arrow indicates the position of the repetitive region. Nter, amino-terminal conserved region; CterI, CterII and CterIII, carboxy-terminal conserved regions; Repetitive, repetitive region.

**Figure 3:**
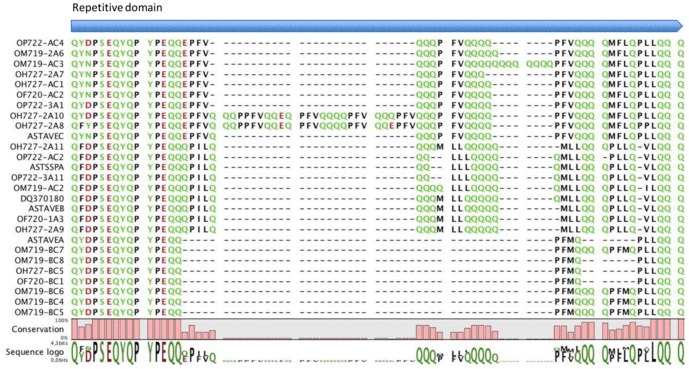
Alignment of the repetitive domain for the avenins protein sequences.

**Figure 4:**
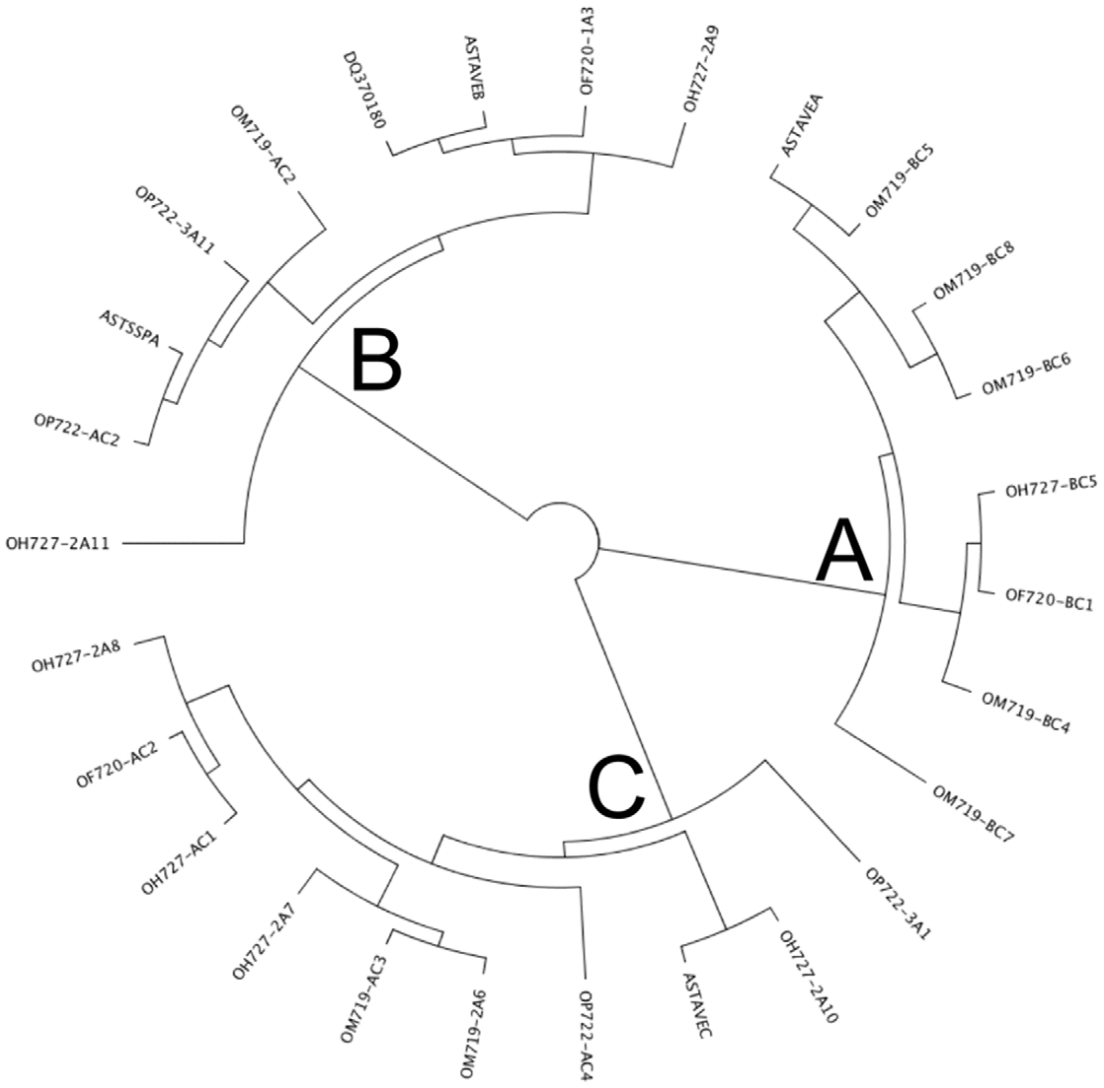
Neighbor-joining tree obtained with an alignment of the avenin sequences shown in the alignment of **Figure 2**. A, B and C indicate the major groups where the sequences are clustered.

All sequences were aligned with the avenin sequences deposited at GenBank (Figures 2 and 3). The avenin sequence alignment comprised a signal peptide followed by five regions or domains. The first was the amino-terminal end. The repetitive region was followed by the last three carboxy-terminal domains containing the cysteine residues. Domain carboxy-terminal II consisted mainly of polyglutamine stretches of variable length. The repetitive domain was characterized by tandem repeats, except in the avenin sequences belonging to group “B” (see next section and Figure 4), where those repeats were absent (Figure 3). The sequences belonging to group “C” clearly showed a peptide repeated in tandem, formed by a core of three residues, giving the peptide PFV, plus a glutamine run of two to four residues (PFV(Q)_2–4_). In the sequences belonging to group “A”, the repeated peptide is more difficult to identify, because it was repeated in only some sequences. This peptide was similar to that of group “C”, with Val replaced by Met in the tripeptide PFV (PFM(Q)_1 or 4_).

### Relatedness of avenin sequences and amino acids composition analysis

Figure 4 shows a neighbor-joining tree constructed using the full protein sequences aligned in Figure 2. The sequences, in the neighbor-joining tree, cluster into three major groups: A, B, and C (Figure 4). All varieties reported in this paper have sequences in groups A, B, and C, except the oat line OP722 which has sequences only in groups B and C.

The average amino acid composition of each prolamin group was calculated using the gliadin, glutenin, and avenin sequences from the GenBank, and the avenin sequences isolated in this paper (Table 5, Figure S3). The 20 amino acids in the prolamin groups were significantly different between oats and wheat. In the wheat gliadins, the major amino acids were Pro, Gln and Leu, except in ω-gliadins with just Pro and Gln comprising 65% of the total amino acids. The wheat glutenins shared as major amino acids Pro, Gln and Ser, but the HMW range also presented Gly and Leu as the major amino acids. The three groups of avenins shared Pro, Gln, Val, and Leu, but avenins from group A also presented Met as a major component. All prolamin groups studied in this work had Pro and Gln as major amino acids. Furthermore, avenins, unlike wheat prolamins, are rich in Val, and wheat glutenins presented Ser as major amino acid, unlike the other groups of prolamins analyzed in this paper.

**Table 5:** Means of frequency percentage of each amino acid calculated from the avenin sequences, reported in this work, and from the sequences of the gluten proteins presents in the GenBank.

		Avenins (%)			Gliadin (%)			Glutenin (%)	
Amino Acid	3-Letter code	A	B	C	ω	α	γ	LMW	HMW
Alanine	Ala	4.87 abc	4.14 bc	5.86 ab	1.36 d	3.81 bc	3.71 c	3.87 bc	6.75 a
Cysteine	Cys	4.87 a	3.69 b	3.72 b	0.14 d	2.06 c	2.74 c	2.33 c	1.97 c
Aspartic acid	Asp	0.54 abc	0.92 abc	0.59 abc	0.21 c	0.38 bc	0.96 ab	0.24 bc	1.13 a
Glutamic acid	Glu	1.62 b	2.31 b	2.5 b	1.95 b	1.75 b	1.04 b	1.73 b	4.29 a
Phenylalanine	Phe	2.43 de	5.25 bc	6.79 ab	7.72 a	3.83 cd	4.57 c	4.55 c	1.92 e
Glycine	Gly	4.33 b	2.58 b	1.16 b	1.09 b	2.51 b	2.76 b	2.77 b	**11.22 a**
Histidine	His	1.62 ab	0.92 cd	0.89 cd	1.06 bcd	2.02 a	1.67 ab	1.37 abc	0.58 d
Isoleucine	Ile	4.33 b	4.31 b	3.15 c	4.69 b	4.89 ab	5.86 a	4.64 b	1.84 d
Lysine	Lys	0.68 b	0 b	0.51 b	0.79 ab	0.6 b	0.97 ab	0.89 ab	2.19 a
Leucine	Leu	**5.96 bc**	**8.89 a**	**7.53 ab**	4.21 c	**8.3 ab**	**7.22 ab**	7.95 ab	**7.99 ab**
Methionine	Met	**7.85 a**	4.08 b	1.77 cd	0.42 d	1.01 cd	2.27 c	2.05 c	1.53 cd
Asparagine	Asn	0.54 bcd	0.51 cd	1.22 b	0.6 bcd	2.41 a	1.99 a	0.82 bc	0.07 d
Proline	Pro	**8.39 c**	**8.67 c**	**9.73 c**	**24.84 a**	**15.04 b**	**15.05 b**	**13.5 b**	**8.44 c**
Glutamine	Gln	**22.24 cd**	**29.25 bc**	**29.25 bc**	**40.18 a**	**32.97 ab**	**31.19 b**	**33.26 ab**	**20.72 d**
Arginine	Arg	3.79 ab	3.02 bc	2.75 bc	1.34 c	1.87 bc	1.74 bc	2.22 bc	5.15 a
Serine	Ser	3.79 cd	3.09 de	2.28 e	4.67 bc	5.22 bc	5.68 b	**8 a**	**7.53 a**
Threonine	Thr	2.71 b	1.92 b	2.04 b	2.91 ab	2.73 b	3.29 ab	3.01 ab	4.64 a
Valine	Val	**6.5 ab**	**6.3 ab**	**8.38 a**	0.67 c	4.99 b	5.23 b	5.11 b	6.9 ab
Tryptophan	Trp	0 c	0 c	0 c	0.08 bc	0.31 abc	0.73 ab	0.48 abc	0.89 a
Tyrosine	Tyr	2.71 bc	1.38 d	1.86 cd	1.07 d	3.31 ab	1.26 d	1.22 d	4.16 a

In bold are indicated the major amino acids. Values within the same row followed by the same letter are not significantly different at *p*<0.05 according to Tukey's HSD (Honestly Significant Difference) test. A, B and C indicate the major avenin groups.

### Analysis of expression patterns of avenin genes of oat cultivars that differ in toxicity for celiac disease by real time PCR

To know the expression patterns of avenin genes during seed development, we analyzed three cultivars of *A. sativa* with different CD-toxicity designated OF720 (non-toxic variety), OH727 (medium-toxic variety) and OM719 (high-toxicity variety), described by Comino et al., 2011. The expression was studied at 0, 4, 8, 12, 20 and 28 DPA, using quantitative RT-PCR with the primer pairs AvenB-q1F/AvenB-q1R, AvenA2-q1F/AvenA2-q1R, and AvenA1-q1F/AvenA1-q1R (Table 1), corresponding to the “A”, “B”, and “C” neighbor-joining tree groups, respectively (Figure 4).

**Figure 5:**
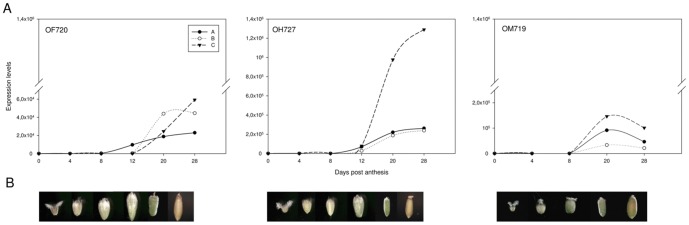
Analysis of avenin genes expression in three cultivars of *A. sativa* during seed development. (A) qRT-PCR analysis of selected avenin groups (A, B and C) was carried out in these varieties. Induction ratios were calculated between the different Days Post Anthesis (DPA) and seeds collected at 0 DPA. Numbers on the x axis indicate the DPA seed, and on the y axis the fold induction of gene expression in relation to the stage 0 DPA. (B) Representative images of seeds collected at 0, 4, 8, 12, 20 and 28 DPA in OF720, OH727 and OM719 cultivars are shown.

The expression of all avenin groups was detectable at 8 DPA in the three different cultivars of *A. sativa* (Figure 5). Varieties OF720 and OH727 presented a continuous increase in expression levels from 8 DPA to 28 DPA, the highest level of expression. However, OM719 cultivar reached the maximum level of expression at 20 DPA, with expression decreasing from that stage of development. We observed that avenins of group “C” were expressed at higher level than those of groups “A” and “B” in all varieties. Avenins of group “B” in OH727 and OM719 cultivars, and avenins of group “A” in OF720, were expressed at the lowest levels. Thus, the expression of avenin genes and therefore the synthesis of avenins, begins at an early stage of grain development and continues even three weeks after anthesis.

### Analysis of immunogenicity of peptides present in prolamins of different oat cultivars

To evaluate whether the distinct CD-toxicity of cultivars OM719 (high-toxic variety), OH727 (medium-toxic variety) and OF720 (non-toxic variety) described previously by Comino et al., 2011 was correlated with the greater or lesser immunogenicity of peptides present in their prolamin sequences, we challenged the synthesized peptides with T-cells obtained from CD patients. The clinical and immunological characteristics of those patients are presented in Table 2.

**Figure 6:**
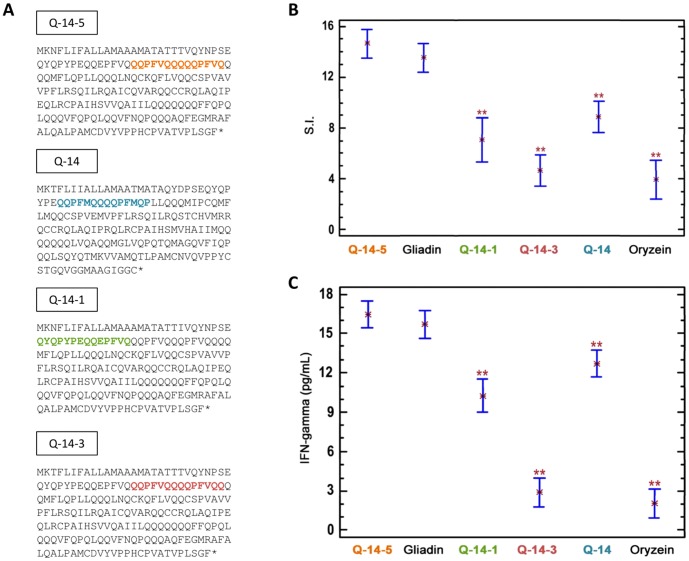
Immunogenicity of different peptides present in three oat varieties. (A), (B) and (C) Avenin sequences with different peptides synthesized for OM719 variety, for OH727 and OF720 varieties and for all cultivars, respectively. (D) Proliferative responses of peripheral blood mononuclear cells (PBMCs) to peptides of prolamins from three different oat varieties. The experiments were performed in duplicate and the mean stimulation index (SI) ±SD is shown. **, -SI values of PBMCs exposed to peptides significantly different with respect to gliadin at p<0.005. (E) Interferon-γ (IFN-γ) production by PBMCs with peptides from three different oat varieties. The results are shown as the means of duplicate wells and expressed as pg/mL. **, significantly different with respect to gliadin at p<0.005. In C and D, gliadin and rice prolamin were used as the positive and negative control, respectively. The color code of peptides used in A, B and C is the same in both C and D.

Immunogenicity of different peptides was determined by T-cell proliferation and IFN-γ production. Peptides were designed based on similarities and differences between the sequences of avenin repetitive region (rich in Pro and Gln) of oat varieties with different toxicity for celiac patients. Four peptides were selected; two (Q-14-5, QQPFVQQQQQPFVQ and Q-14, QQPFMQQQQPFMQP) present in avenin sequences of OM719 cultivar and absent in OH727 and OF720 cultivars (Figure 6A), another present in OH727 and OF720 varieties and absent in OM719 cultivar (Q-14-3, QQPFVQQQQPFVQQ) (Figura 6B) and one present in all varieties (Q-14-1, QYQPYPEQQEPFVQ) (Figure 6C). We tested whether there was a correlation between the immunotoxicity of each variety and the potential immunotoxicity of peptides present in their sequences for CD patients.

Cell proliferation in the culture medium were measured as indices of PBMC activation. Cell proliferation and IFN-γ release in the culture medium were measured as indexes of PBMCs activation. We found a significant increase difference with respect to gliadin (positive control) in T-cell proliferation in cultures incubated with Q-14 and Q-14-1 peptides with proliferation index of 9.2±0.7 and 6.7±0.9, respectively (Figure 6C). The incubation with Q-14-3 peptide increased cell proliferation (SI = 4.1±0.3) similarly to that of incubation with rice prolamin used as negative control (SI = 3.5±0.2), being less immunogenic. The highest increase in T-cell proliferation was found in cultures incubated with Q-14-5 peptide (SI = 17+0.9), even more elevated than that produced by gliadin although this increase was not significant. These results clearly showed that Q-14-5 peptide displayed the highest activity, and was the most potentially immunogenic (Figure 6C).

Release of IFN-γ in the culture medium after the exposure of celiac PBMCs to peptides was assessed (Figure 6E). According to this assay, gliadin and Q-14-5 peptide were very immunogenic with the highest values of IFN-γ release (16±1.4 pg/mL and 17.5±1.2 pg/mL, respectively). We found significant differences with respect to positive control in the exposure to Q-14 peptide, which induced a lower mean value of IFN-γ (12±0.9 pg/mL), similarly to the exposure with Q-14-1 peptide (9.5±0.5 pg/mL). Finally, Q-14-3 peptide was the least immunogenic (2.9±0.2 pg/mL), with IFN-γ levels similar to those of rice prolamin.

## Discussion

The prolamins of the tribe Triticeae (wheat, rye, and barley) are classified according to amino acid sequence into three different types of protein: sulfur-rich (S-rich), sulfur-poor (S-poor) and high molecular weight (HMW) prolamins, with several subgroups within the S-rich group [26].

Avenin proteins (alcohol-soluble fraction) have structural homology to the S-rich subgroup α-gliadins and γ-gliadins of wheat, the B-hordeins of barley, and the γ-secalins of rye [27]. Our results agree with the cited homology analysis, because the oat gliadin-like chromatogram shows major peaks at the same retention time ranges as α- and γ-gliadins from the wheat control, with only minor peaks in the hydrophilic region corresponding to the ω-gliadins. Herbert Wieser & H.-D. Belitz (1989) reported that the minor peaks in the hydrophilic region are contaminations from non-prolamin proteins [28].

Once the gliadin-like (alcohol-soluble) proteins from oat flour had been extracted, the remaining pellet was extracted again with a denaturing/reducing solution to obtain a protein fraction similar to wheat glutenin. The HPLC chromatogram of the extract showed minor peaks in the hydrophilic region, in the retention time range of HMW-GS from wheat. In contrast, major peaks appeared with the same retention time as the LMW-GS from the wheat control. This confirmed that oat grain contains a significant fraction that is insoluble in alcohol and soluble in denaturing/reducing solution. This fraction had been called “glutelin fraction” by other authors [29]–[31]. Comparison of the chromatograms of glutenin-like and gliadin-like fractions from the same oat line indicates that the peaks appearing in the glutenin-like fraction are not contamination from the previously extracted alcohol-soluble fraction. Therefore, we can conclude that oat contains a glutenin-like fraction which contains mainly LMW-GS-like proteins.

The oat lines showed differences in their chromatograms, due to the presence of different prolamin subunits, that allowed differentiation of all the varieties studied in this paper. These results were consistent with previous results reported by Comino et al., 2011 using MALDI-TOF MS and SDS-PAGE. Varietal identification of oats has been clearly demonstrated by RP-HPLC [31]–[33]. Our results confirm that RP-HPLC is an excellent technique for oat cultivar differentiation. Moreover, this technique could be used to identify specific RP-HPLC pattern related to CD toxicity, to select oat varieties with a reduced immunogenicity.

Total protein content in oat kernels typically ranges between 15%-and 20% of total weight [31]. In the present study, prolamins have been shown to represent around 2% of the total weight. Consequently, assuming some 17% of total protein in oat grains, total prolamin (gliadin-like and glutenin-like) content mayrange between 12%-and 14% of the total protein in the oat lines reported in this paper. This result confirms the prolamin content estimated by other authors, who reported that only 10–15% of the total grain proteins in oats are prolamins [6], [34].

The reported avenin sequences present features similar to those of the S-rich prolamins, especially to the γ-gliadin from wheat [6], [35]–[38]. The positions of cysteine residues and the flanking sequences are well conserved in the avenin sequences as in S-rich prolamins [38]. The cysteine residues are responsible for forming intermolecular and intramolecular disulfide linkages during the processing of the grains [39]. Avenin sequences belonging to groups “B” and “C” present eight cysteine residues, whereas avenins of group “A” show nine. A few authors have proposed that some gliadins with an odd number of cysteine residues may also be incorporated into the glutenin polymer [40]–[42]. Later, evidence was provided that a naturally mutated γ-gliadin with an extra cysteine residue is incorporated into the polymeric fraction [43]. It is quite possible that the avenins of group “A” form interchain disulfide bonds and thereby a polymer similar to wheat glutenins. This polymer, linked by disulfide bonds, could consist either of proteins only from group “A”, or of proteins from group “A” together with proteins from other groups or even with other prolamins which have not yet been identified. Therefore, the protein product of avenins from group “A” could be responsible for the RP-HPLC fraction designated LMW-GS-like in this paper. This fraction (LMW-GS-like) must form a polymer, as it was not extracted with the alcoholic solution, but when we used a highly reducing solution, which breaks disulfide bonds, protein constituents were released. We may infer that the protein product of the avenin groups “B” and “C” can lead to the protein fractions termed α- and γ-gliadin-like in the RP-HPLC analysis of the oat proteins, and that avenin of group “A” corresponds with the LMW-GS-like fraction.

The avenins described in this work included among their major amino acids Pro and Gln, which are characteristic of all cereal prolamins [6], [44]. In fact, the name prolamin was originally based on the observation that they are generally rich in Pro and amide nitrogen derived from Gln. In the avenin groups reported here, the combined proportions of these two amino acids vary from 30.6 to 39.0%. These proportions are in accord with the range (30–70%) of the combined proportions of these amino acids found among different cereals and prolamin groups [26]. It is well-known that the Pro content is positively correlated with the celiac toxicity of the storage proteins of various cereals [45]–[47]. The analysis of amino acids content of avenins reported throughout this article shows that avenins have a proline content that is lower than wheat gliadins and LMW glutenin subunits, and similar to HMW glutenin subunits. Therefore, the lower Pro content of avenins could determine the lower celiac toxicity with respect to wheat prolamins.

Knowing the expression patterns of different storage proteins, as well as the features of their sequences, is important because they determine the characteristics of mature grain and possibly the potential immunogenicity for CD patients. In this work, we have used qRT-PCR, a specific method to gene quantification, to study the expression of avenin genes in different cultivars of *A. sativa* that differ in their toxicity for CD patients. This method is 1000-fold more sensitive than dot blot hybridization, it can discriminate between messenger RNAs (mRNAs) with similar sequences, and it needs a lesser amount of RNA template than do other methods of gene expression [48]. To date, the expression of avenin genes has not been extensively studied, so there is no accepted pattern of expression. Some authors have described early expression patterns where avenins begin to be detectable between 4 DPA and 6 DPA, reaching the maximum at 8 DPA [27]. In contrast, other researchers accept a later expression pattern in which the maximum accumulation of avenins occurs two weeks after anthesis [49]. We have shown that the expression of avenin genes begins at 8 DPA, and reach the maximum level of expression between 20 DPA and 28 DPA. Thus expression begins at early stages of seed development and continues until late stages, when the peak is reached. Hence, oats follow an expression pattern similar to that in wheat and other cereals [38], [50]. Furthermore, according to the results obtained by RP-HPLC, we have observed greater expression of α- and γ-gliadin-like (avenins group “C”) than of LMW-GS-like (avenins of group A).

In this work, we have shown by *in vitro* studies a direct correlation of the immunogenicity of the different oat varieties with the toxicity of peptides present in their avenin sequences. In fact, the Q-14-5 peptide present in avenin sequences of the OM719 variety is potentially immunotoxic. However, the Q-14-3 peptide from OH727 and OF720 proved to be non-active in triggering both T-cell proliferation and IFN-γ release, showing similar values to the negative control. Furthemore, the peptide present in all cultivars (Q-14-1) was not immunotoxic either. These results suggest that the differences in the protein sequences of different oat cultivars could explain why certain varieties of oats are toxic for CD patients and other not.

## Supporting Information

Figure S1 **Major peak details of RP-HPLC chromatograms of oat gliadin-like fractions.** The major peaks were designated from “a” to “q” according to their retention time. mAU (210 nm), milliunits of absorbance at 210 nm; min, retention time in minutes.
(TIFF)

Figure S2 **Comparison of the RP-HPLC chromatograms of gliadin-like and glutenin-like fractions from OP722.** mAU (210 nm), milliunits of absorbance at 210 nm; min, retention time in minutes.
(TIFF)

Figure S3 **Means of frequency percentage of each amino acid calculated from the avenin sequences, reported in this work, and from the sequences of the gluten proteins presents in the GenBank.** av.a, avenins form group A; av.b, avenins form group B; av.c, avenins form group C; o, ω.gliadins; a, α-gliadins; g, γ-gliadins; L, LMW-glutenin subunits; H, HMW-glutenin subunits. The 95% confidence interval is also included.
(TIFF)

## References

[1] West J, Logan RF, Card TR, Smith C, Hubbard R (2003) Fracture risk in people with celiac disease: a population-based cohort study. Gastroenterology 125: 429–436. 12891545 10.1016/s0016-5085(03)00891-6

[2] Wieser H, Koehler P (2008) The biochemical basis of celiac disease. Cereal Chem 85: 1–13.

[3] Sollid LM (2002) Coeliac disease: dissecting a complex inflammatory disorder. Nat Rev Immunol 2: 647–655. 12209133 10.1038/nri885

[4] Osborne TB (1908) Our present knowledge of plant proteins. Science 28: 417–427. 17771936 10.1126/science.28.718.417

[5] Wieser H, Antes S, Seilmeier W (1998) Quantitative determination of gluten protein types in wheat flour by reversed-phase high-performance liquid chromatography. Cereal Chem 75: 644–650.

[6] Shewry PR, Napier JA, Tatham AS (1995) Seed storage proteins: structures and biosynthesis. Plant Cell 7: 945–956. 7640527 10.1105/tpc.7.7.945 PMC160892

[7] Stern M, Ciclitira PJ, van Eckert R, Feighery C, Janssen FW, et al (2001) Analysis and clinical effects of gluten in coeliac disease. Eur J Gastroenterol Hepatol 13: 741–747. 11434606 10.1097/00042737-200106000-00023

[8] Morón B, Bethune MT, Comino I, Manyani H, Ferragud M, et al (2008) Toward the assessment of food toxicity for celiac patients: characterization of monoclonal antibodies to a main immunogenic gluten peptide. PLoS One 3: e2294. 18509534 10.1371/journal.pone.0002294 PMC2386552

[9] Morón B, Cebolla Á, Manyani H, Álvarez-Maqueda M, Megías M, et al (2008) Sensitive detection of cereal fractions that are toxic to celiac disease patients by using monoclonal antibodies to a main immunogenic wheat peptide. Am J Clin Nutr 87: 405–414. 18258632 10.1093/ajcn/87.2.405

[10] Shan L, Molberg Ø, Parrot I, Hausch F, Filiz F, et al (2002) Structural basis for gluten intolerance in celiac sprue. Science 297: 2275–2279. 12351792 10.1126/science.1074129

[11] Suttie JM, Reynolds SG eds (2004) Fodder oats: a world overview. Food and Agriculture Organization of the United Nations (FAO). Rome. (FAO website. Available: http://www.fao.org/docrep/008/y5765e/y5765e00.htm. Accessed 2012 October 16.).

[12] Janatuinen EK, Pikkarainen PH, Kemppainen TA, Kosma VM, Järvinen RM, et al (1995) A comparison of diets with and without oats in adults with celiac disease. N Engl J Med 333: 1033–1037. 7675045 10.1056/NEJM199510193331602

[13] Brennan CS, Cleary LJ (2005) The potential use of cereal (1→3,1→4)-β-d-glucans as functional food ingredients. J Cereal Sci 42: 1–13.

[14] Eppendorfer WH (2006) Nutritive value of oat and rye grain protein as influenced by nitrogen and amino acid composition. J Sci Food Agric 28: 152–156. 10.1002/jsfa.2740280207 853720

[15] Arentz-Hansen H, Fleckenstein B, Molberg Ø, Scott H, Koning F, et al (2004) The molecular basis for oat intolerance in patients with celiac disease. PLoS Med 1: e1. 15526039 10.1371/journal.pmed.0010001 PMC523824

[16] Pulido OM, Gillespie Z, Zarkadas M, Dubois S, Vavasour E, et al (2009) Introduction of oats in the diet of individuals with celiac disease: A Systematic Review. Adv Food Nutr Res 57: 235–285. 19595389 10.1016/S1043-4526(09)57006-4

[17] Comino I, Real A, de Lorenzo L, Cornell H, López-Casado MÁ, et al (2011) Diversity in oat potential immunogenicity: basis for the selection of oat varieties with no toxicity in coeliac disease. Gut 60: 915–922. 21317420 10.1136/gut.2010.225268 PMC3112367

[18] Pistón F, Gil-Humanes J, Rodríguez-Quijano M, Barro F (2011) Down-regulating γ-gliadins in bread wheat leads to non-specific increases in other gluten proteins and has no major effect on dough gluten strength. PLoS ONE 6: e24754. 21935456 10.1371/journal.pone.0024754 PMC3172295

[19] Team RDC (2012) R: A Language and Environment for Statistical Computing. Vienna, Austria. (R project website. Available: http://www.R-project.org/. Accessed 2012 October 16.).

[20] Fox J, Weisberg S (2011) An R Companion to Applied Regression. Second. Thousand Oaks CA: Sage. (Sage Publications website. Available: http://socserv.mcmaster.ca/jfox/Books/Companion/index.html. Accessed 2012 October 16.).

[21] Mendiburu F de (2012) agricolae: Statistical Procedures for Agricultural Research. (CRAN website. Available: http://CRAN.R-project.org/package=agricolae. Accessed 2012 October 16.).

[22] Bolker GRWIR source code and/or documentation contributed by B, Bonebakker L, Gentleman R, Liaw WHA, Lumley T, et al. (2009) gplots: Various R programming tools for plotting data. (CRAN website. Available: http://CRAN.R-project.org/package=gplots. Accessed 2012 Oct 16.)

[23] Zadoks JC, Chang TT, Konzak CF (1974) A decimal code for the growth stages of cereals. Weed Res 14: 415–421.

[24] Marsh MN, Crowe PT (1995) 5 Morphology of the mucosal lesion in gluten sensitivity. Baillière's Clin Gastroenterol 9: 273–293. 7549028 10.1016/0950-3528(95)90032-2

[25] Burnouf T, Bietz JA (1985) Chromosomal control of glutenin subunits in aneuploid lines of wheat: analysis by reversed-phase high-performance liquid chromatography. Theor Appl Genet 70: 610–619. 24253118 10.1007/BF00252286

[26] Shewry PR, Halford NG (2002) Cereal seed storage proteins: structures, properties and role in grain utilization. J Exp Bot 53: 947–958. 11912237 10.1093/jexbot/53.370.947

[27] Chesnut RS, Shotwell MA, Boyer SK, Larkins BA (1989) Analysis of avenin proteins and the expression of their mRNAs in developing oat seeds. Plant Cell 1: 913–924. 2535531 10.1105/tpc.1.9.913 PMC159827

[28] Wieser H, Belitz H-D (1989) Amino acid compositions of avenins separated by reversed-phase high-performance liquid chromatography. Journal of Cereal Science 9: 221–229.

[29] Wieser H, Seilmeier W, Belitz HD (1980) Vergleichende Untersuchungen über partielle Aminosäuresequenzen von Prolaminen und Glutelinen verschiedener Getreidearten. Zeitschrift für Lebensmitteluntersuchung und-Forschung A 170: 17–26.

[30] Dvořáček V, Čurn V, Moudry` J (2003) Suitability of oat-seed storage-protein markers for identification of cultivars in grain and mixed flour samples. Plant Soil Environ 49: 486–491.

[31] Lapvetelainen A, Bietz J, Huebner F (1995) Reversed-phase high-performance liquid chromatography of oat proteins: application of cultivar comparison and analysis of the effect of wet processing. Ceral Chem 72: 259–264.

[32] Lookhart G (1985) Identification of oat cultivars by combining polyacrylamide gel electrophoresis and reversed-phase high-performance liquid chromatography. Cereal Chem 62: 345–350.

[33] Lookhart G, Pomeranz Y (1985) Characterization of oat species by polyacrylamide gel electrophoresis and high performance liquid chromatography of their prolamin proteins. Cereal Chem 62: 162–166.

[34] Lamacchia C, Chillo S, Lamparelli S, Suriano N, La Notte E, et al (2010) Amaranth, quinoa and oat doughs: Mechanical and rheological behaviour, polymeric protein size distribution and extractability. J Food Eng 96: 97–106.

[35] Forde J, Malpica JM, Halford NG, Shewry PR, Anderson OD, et al (1985) The nucleotide sequence of a HMW glutenin subunit gene located on chromosome 1A of wheat (*Triticum aestivum* L.). Nucleic Acids Res 13: 6817. 2997729 10.1093/nar/13.19.6817 PMC322007

[36] D'Ovidio R, Tanzarella OA, Porceddu E (1991) Cloning and sequencing of a PCR amplified gamma-gliadin gene from durum wheat (*Triticum turgidum* (L.) Thell. conv. durum (Desf.) MK.). Plant Sci 75: 229–236.

[37] Pistón F, Dorado G, Martín A, Barro F (2004) Cloning and characterization of a γ-3 hordein mRNA (cDNA) from *Hordeum chilense* (Roem. et Schult.). Theor Appl Genet 108: 1359–65. 14747917 10.1007/s00122-003-1548-x

[38] Pistón F, Dorado G, Martín A, Barro F (2006) Cloning of nine γ-gliadin mRNAs (cDNAs) from wheat and the molecular characterization of comparative transcript levels of γ-gliadin subclasses. J Cereal Sci 43: 120–128.

[39] Shewry PR, Miflin BJ, Kasarda DD (1984) The structural and evolutionary relationships of the prolamin storage proteins of barley, rye and wheat. Philos Trans R Soc Lond B Biol Sci 304: 297–308.

[40] D'Ovidio R, Masci S (2004) The low-molecular-weight glutenin subunits of wheat gluten. J Cereal Sci 39: 321–339.

[41] Gianibelli M, Larroque O, MacRitchie F, Wrigley C (2001) Biochemical, genetic, and molecular characterization of wheat glutenin and its component subunits. Cereal Chem 78: 635–646.

[42] Masci S, Egorov T, Ronchi C, Kuzmicky D, Kasarda D, et al (1999) Evidence for the presence of only one cysteine residue in the D-type low molecular weight subunits of wheat glutenin. J Cereal Sci 29: 17–25.

[43] Ferrante P, Masci S, D'Ovidio R, Lafiandra D, Volpi C, et al (2006) A proteomic approach to verify in vivo expression of a novel γ-gliadin containing an extra cysteine residue. Proteomics 6: 1908–1914. 16485260 10.1002/pmic.200500236

[44] Shewry PR, Tatham AS (1990) The prolamin storage proteins of cereal seeds: structure and evolution. Biochem J 267: 1–12. 2183790 10.1042/bj2670001 PMC1131235

[45] Wieser H (1996) Relation between gliadin structure and coeliac toxicity. Acta Paediatr 85: 3–9. 10.1111/j.1651-2227.1996.tb14239.x 8783747

[46] Detlef S (2000) Current concepts of celiac disease pathogenesis. Gastroenterology 119: 234–242. 10889174 10.1053/gast.2000.8521

[47] Hausch F, Shan L, Santiago NA, Gray GM, Khosla C (2002) Intestinal digestive resistance of immunodominant gliadin peptides. Am J Physiol Gastrointest Liver Physiol 283: G996–G1003. 12223360 10.1152/ajpgi.00136.2002

[48] Wong ML, Medrano JF (2005) Real-time PCR for mRNA quantitation. Biotechniques 39: 75. 16060372 10.2144/05391RV01

[49] Robert LS, Nozzolillo C, Altosaar I (1983) Molecular weight and charge heterogeneity of prolamins (Avenins) from nine oat (*Avena sativa* L.) cultivars of different protein content and from developing seeds. Cereal Chem 60: 438–442.

[50] Han Z, Wu F, Deng G, Qian G, Yu M, et al (2010) Structural and expressional analysis of the B-hordein genes in Tibetan hull-less barley. Genetica 138: 227–239. 19856114 10.1007/s10709-009-9415-6

