# Supplementary figures and images for: Molecular and Immunological Characterization of Gluten Proteins Isolated from Oat Cultivars That Differ in Toxicity for Celiac Disease
Source: PLoS One. 2012 Dec 17;7(12):e48365. doi: 10.1371/journal.pone.0048365 (PMC3524229; doi:10.1371/journal.pone.0048365)

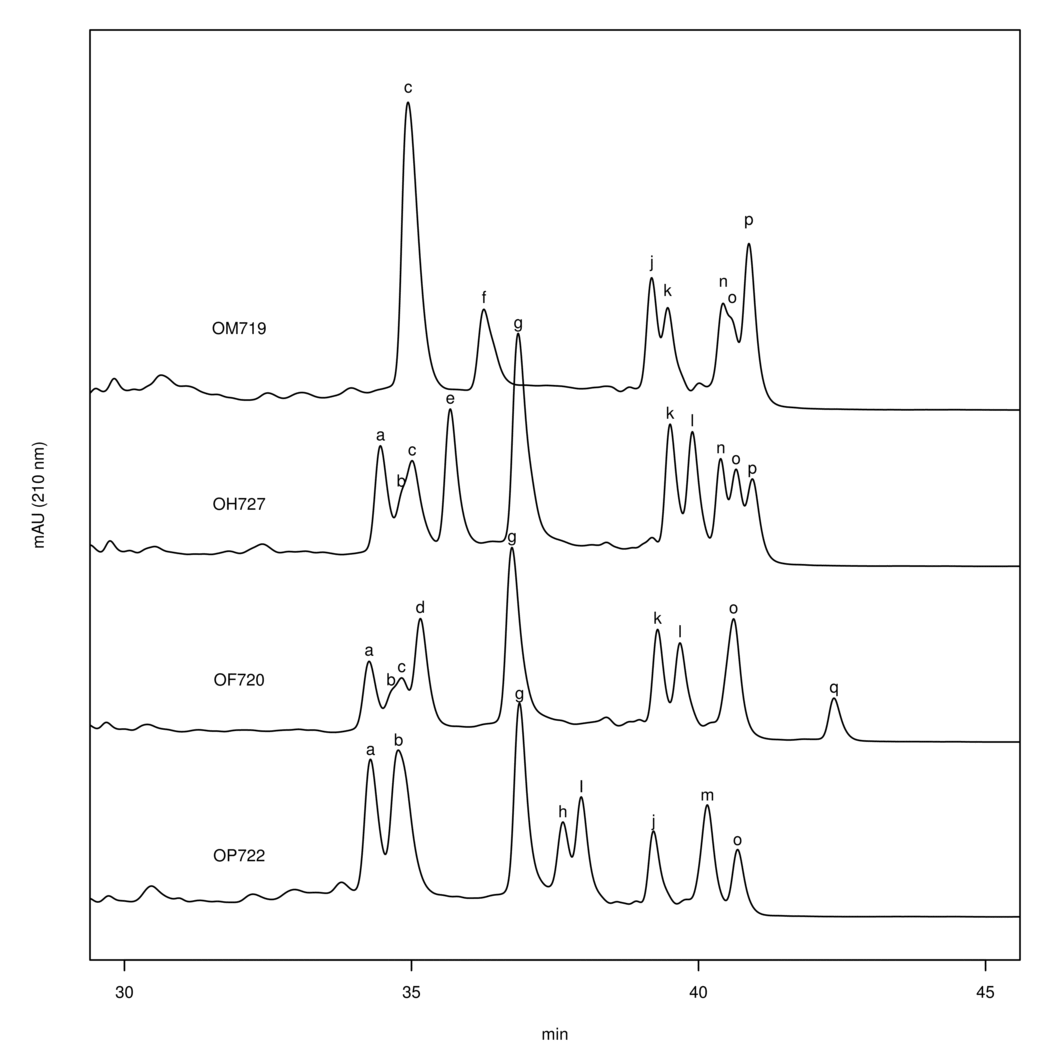

Supplement: Figure S1 — Major peak details of RP-HPLC chromatograms of oat gliadin-like fractions. The major peaks were designated from “a” to “q” according to their retention time. mAU (210 nm), milliunits of absorbance at 210 nm; min, retention time in minutes. (TIFF) [file pone.0048365.s001.tiff]

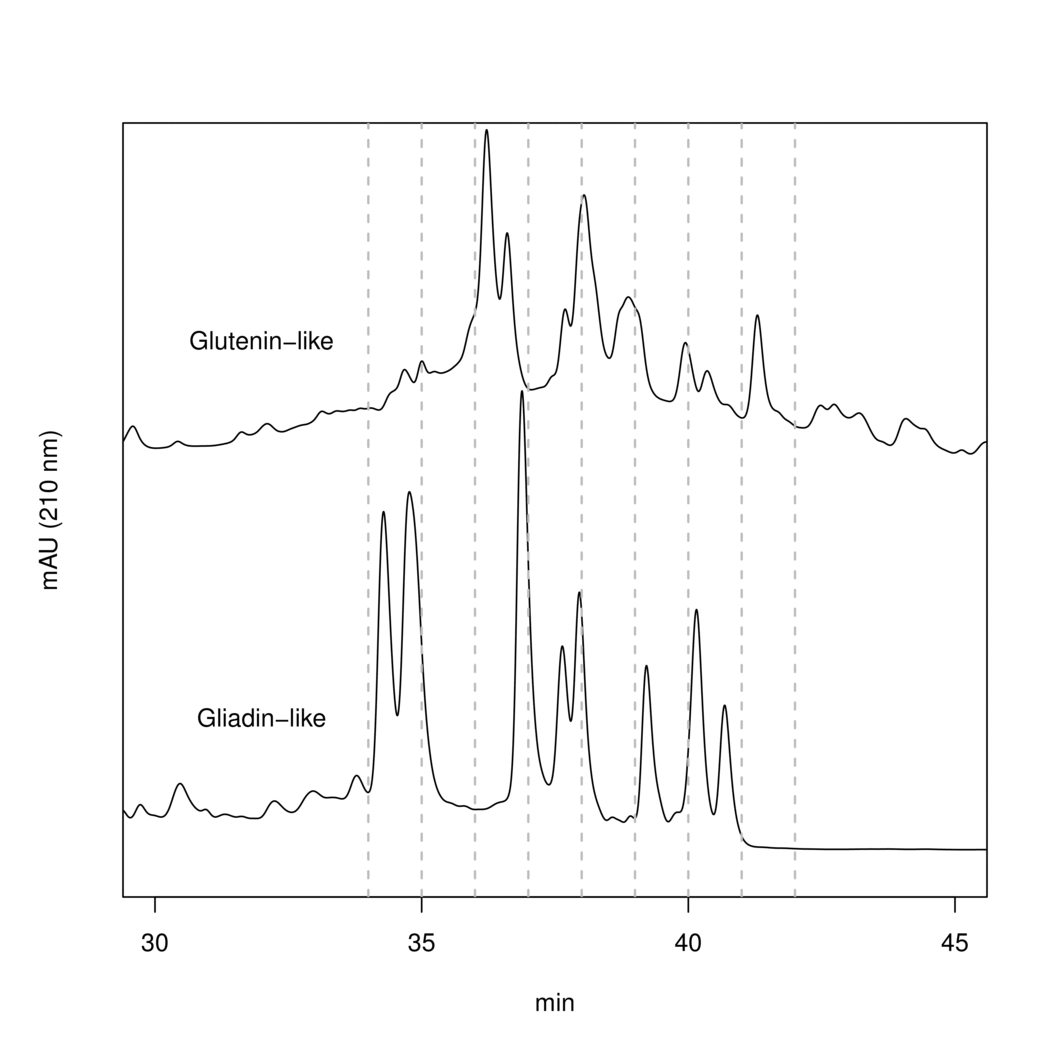

Supplement: Figure S2 — Comparison of the RP-HPLC chromatograms of gliadin-like and glutenin-like fractions from OP722. mAU (210 nm), milliunits of absorbance at 210 nm; min, retention time in minutes. (TIFF) [file pone.0048365.s002.tiff]

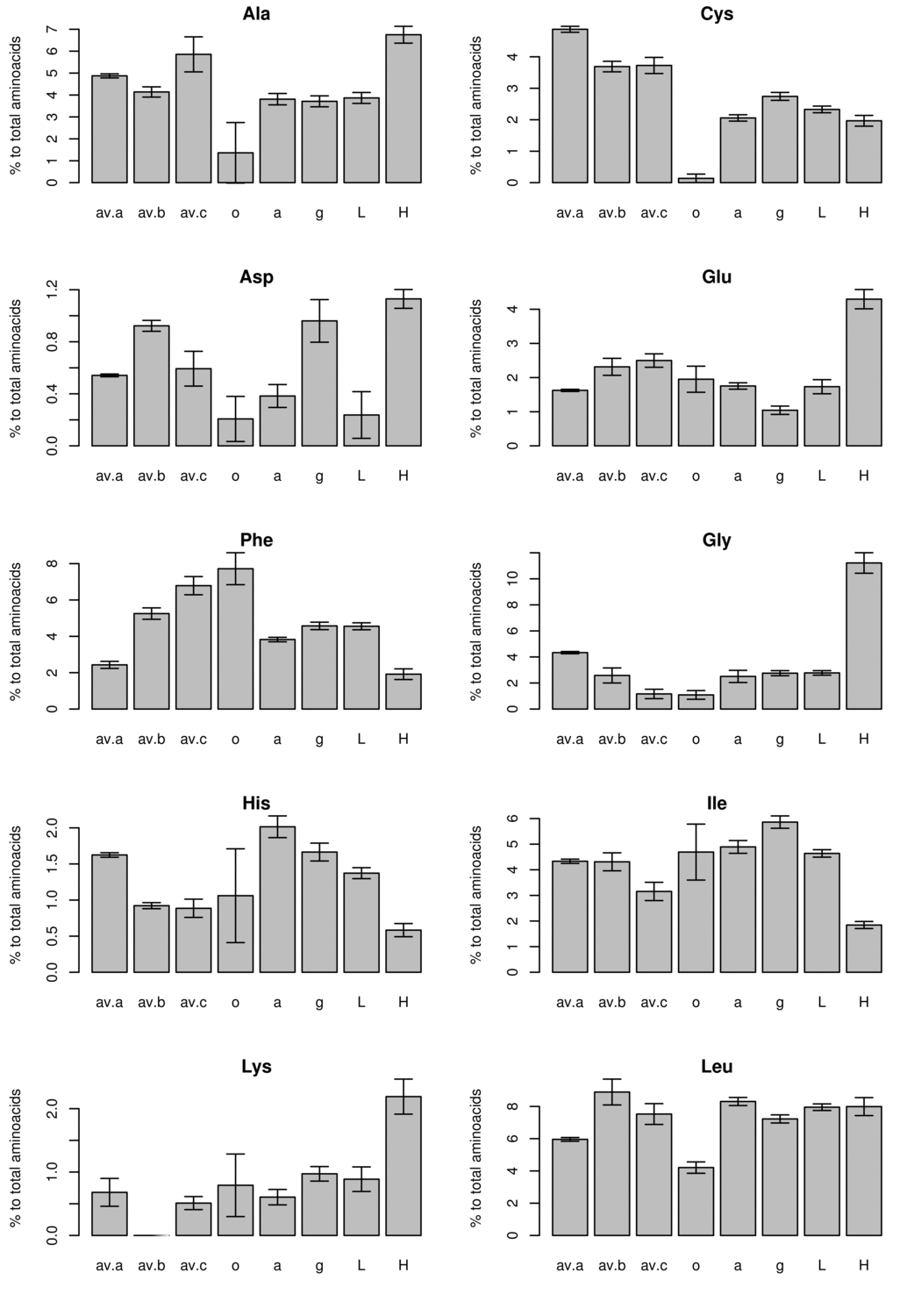

Supplement: Figure S3 — Means of frequency percentage of each amino acid calculated from the avenin sequences, reported in this work, and from the sequences of the gluten proteins presents in the GenBank. av.a, avenins form group A; av.b, avenins form group B; av.c, avenins form group C; o, ω.gliadins; a, α-gliadins; g, γ-gliadins; L, LMW-glutenin subunits; H, HMW-glutenin subunits. The 95% confidence interval is also included. (TIFF) [file pone.0048365.s003.tiff]
